# Stewarding scarce response capacity: an inductive qualitative interview study of emergency medical dispatchers’ prioritising ambulance resources

**DOI:** 10.1136/bmjopen-2026-118269

**Published:** 2026-07-02

**Authors:** Peter Hill, Jakob Lederman, Daniel Jonsson, Peter Bolin, Veronica Vicente

**Affiliations:** 1 Department of Clinical Science and Education, Karolinska Institutet, Stockholm, Sweden; 2 Health and Medical Care Administration, Department for Specialized Care, Stockholm, Sweden; 3 Department of Clinical Science and Education, Södersjukhuset, Karolinska Institutet, Stockholm, Sweden; 4 Urban Planning and Environment, KTH, Stockholm, Sweden; 5 Learning, Informatics, Management and Ethics, Karolinska Institutet, Stockholm, Sweden; 6 The Ambulance Medical Service in Stockholm, Stockholm, Sweden; 7 Academic EMS, Stockholm, Sweden

**Keywords:** Emergency Service, Hospital, QUALITATIVE RESEARCH, Decision Making

## Abstract

**Abstract:**

**Objective:**

This study aimed to explore emergency medical dispatchers’ (EMDs’) experiences of prioritising patients and stewarding ambulance resources when system capacity was constrained.

**Design:**

Qualitative interview study using inductive qualitative content analysis.

**Setting:**

Emergency medical communication centres (EMCCs) in Sweden, operated by the national emergency call provider and responsible for receiving 112 calls and dispatching ambulances.

**Participants:**

13 purposively sampled EMDs with at least 1 year of professional experience.

**Data analysis:**

Interviews were analysed inductively using qualitative content analysis (Elo and Kyngäs) through open coding, grouping into subcategories and abstraction into generic categories and one main category.

**Results:**

Dispatchers described prioritisation under scarcity as system work that simultaneously addressed individual patient acuity and population-level readiness. One main category captured this work: stewarding scarce response capacity. Three inter-related generic categories characterised stewardship: (1) prioritising by clinical urgency within geographic and operational constraints; (2) producing availability through anticipation, reassessment and queue governance in a ‘virtual waiting room’; and (3) coordinating response through information infrastructures and interprofessional collaboration. Across categories, dispatchers described redistributing risk across patients and time, managing moral strain when delays could harm patients and using experience, reassessment and teamwork to avoid both under-response to urgent need and over-allocation that would leave areas without coverage.

**Conclusions:**

Dispatch under scarcity is best understood as active stewardship of a safety-critical dispatch queue. Strengthening patient safety therefore requires organisational support for reassessment and escalation during prolonged waits, explicit governance of queue dynamics and geographic coverage trade-offs, safeguards for contextual judgement when automation is used and support for dispatchers exposed to morally difficult scarcity decisions.

**STRENGTHS AND LIMITATIONS OF THIS STUDY**

- An inductive qualitative content analysis allowed categories to emerge from dispatchers’ own descriptions, rather than imposing predefined theoretical frameworks.
- Inclusion of emergency medical dispatchers with varied ages, professional experience, occupational backgrounds and broad emergency medical communication centre locations enhanced the richness of the data and potential transferability.
- Analyst triangulation, an explicit abstraction pathway and data-to-category quotations strengthened analytic transparency and trustworthiness.
- Interviews were conducted via video, which may have limited access to non-verbal cues compared with in-person interviews.
- The study was conducted within a single national dispatch system and participation was voluntary, which may limit transferability and introduce self-selection of more experienced or engaged dispatchers.

## Introduction

 Emergency medical communication centres (EMCCs) constitute the first formal link in the prehospital emergency care chain. They receive emergency calls, establish the nature and urgency of incidents and coordinate appropriate responses. In Sweden, calls to the national emergency number (112) are handled by the national provider, which connects callers to emergency medical dispatch. Through early identification of time-critical conditions and timely allocation of resources, EMCCs influence access to emergency care and patient outcomes.^1^

Emergency medical dispatchers (EMDs) verify incident location, elicit salient clinical information and apply structured dispatch protocols to assign a priority level and response type.^2 3^ Their work encompasses call-taking, clinical assessment and the allocation and dispatch of ambulances. This study focuses specifically on the allocation and dispatch component. Priority levels constitute a shared operational language for urgency (eg, immediate responses with lights and sirens, urgent responses without lights and sirens and lower-priority responses that may be safely deferred). However, dispatch is not solely a matter of clinical categorisation. Response decisions are continuously shaped by the real-time availability and geographic distribution of ambulances and other resources, as well as the need to preserve system coverage for sudden high-acuity events.^4 5^

Resource shortages have become a recurrent operational condition in many emergency medical services (EMS) systems, driven by rising demand, prolonged ambulance turnaround times and workforce constraints. When demand exceeds capacity, dispatchers must allocate resources across multiple patients who compete for attention and response. Delays can expose patients to harm through deterioration while waiting, whereas dispatching distant units or over-committing local resources can increase vulnerability elsewhere by leaving geographic areas without immediate response capacity.^6^

Existing research has largely examined scarcity through quantitative indicators such as response times, unit hour utilisation and models for optimal ambulance placement. These approaches can describe system behaviour but provide limited insight into the practical and ethical reasoning required when dispatchers must decide who can safely wait, which risks are acceptable, and how to maintain readiness across a region. Qualitative research has shown that dispatcher decision-making is influenced by call complexity, stress, organisational support and the quality of information elicited during emergency calls.^7 8^ Yet much of this research focuses on call assessment or protocol use, rather than on the broader work of coordinating allocation and maintaining system readiness during sustained scarcity.^4 5 9 10^

An inductive qualitative approach can clarify how dispatchers themselves describe and make sense of prioritisation under scarcity, including how they integrate protocol categories with contextual judgement, ethical responsibility and experiential knowledge. Such knowledge may inform training, organisational support and decision-support tool development that aligns with the realities of dispatch work,^11^ including the governance of queued cases and the coordination of multiple actors.

## Aim

To explore EMDs’ experiences of ambulance resource shortages and patient prioritisation using an inductive qualitative approach.

## Methods

### Study design and reporting

We conducted a qualitative descriptive interview study. Semistructured interviews were used for data collection, and inductive qualitative content analysis was used to analyse interview transcripts. This manuscript follows the qualitative content analysis process described by Elo and Kyngäs^12^ (preparation, organising and reporting phases) and is reported in accordance with the Consolidated criteria for Reporting Qualitative research.^13^

### Setting

The study was conducted within the Swedish emergency medical dispatch system. SOS Alarm AB is commissioned to receive and forward urgent calls to the national emergency number 112 and to assist municipalities and regions with ambulance prioritisation and dispatch.^14 15^ In 2025, SOS Alarm operated 15 SOS centres across Sweden, from Luleå in the north to Malmö in the south. Call-taking for 112 is handled nationally, whereas ambulance dispatch and rescue operations are handled locally, which means that dispatchers work with local and regional resource availability while being embedded in a national emergency call infrastructure.^15^

Catchment areas for ambulance dispatch are primarily defined by regional ambulance service agreements and county/region ambulance service areas, but resources may be coordinated across borders when regional agreements or operational need allow. Ambulance services are delivered by both public and private EMS providers. In addition to 112, members of the public may seek non-emergency health advice through 1177 by telephone or online, contact primary care or out-of-hours services or self-present to emergency departments; however, acute or life-threatening conditions should be directed to 112.^16^ These alternative pathways form part of the wider urgent care landscape but were not the object of analysis in this study, which focused on ambulance dispatch once a request for ambulance response had entered the EMCC workflow.

Ambulance dispatch is guided by a national criteria-based decision support system and by regional dispatch rules. During the study period, participants described priority levels as a shared operational language: priority 1A denoted immediately life-threatening conditions requiring the fastest possible response; priority 1B denoted other life-threatening or potentially life-threatening conditions requiring immediate response; priority 2A denoted urgent conditions requiring prompt ambulance response but normally without lights and sirens; priority 2B denoted urgent conditions in which dispatchers could take greater account of resource and coverage conditions; priority 3 denoted lower-acuity cases or transports that could be deferred; and priority 4 or lying medical transport was used in some regions for lower-acuity transport needs. Exact response-time targets and resource rules varied by region. Therefore, these definitions are presented functionally rather than as universal national performance standards.

Operational EMCC teams usually include 112 operators or call-takers, ambulance dispatchers, registered nurses or medical advisors, personnel in rescue-service dispatch roles and, in some centres or shifts, internal officers or operational coordinators who support system-level ambulance resource coordination.^17^ Operators and dispatchers complete internal operational training and may have varied occupational backgrounds; in this study participants’ prior backgrounds included healthcare, rescue service and non-healthcare service work. Registered nurses or medical advisors provide clinical support, reassessment and escalation support in selected cases. Teamwork is therefore partly formalised through roles, shared information systems, call-monitoring functions and regional routines and partly informal through peer-to-peer micro-coordination during high workload.

In this manuscript, ‘internal officer’ (IB) denotes operational staff involved in ambulance resource coordination. The term 'virtual waiting room’ is used as an analytic shorthand grounded in participants’ use of the Swedish term 'väntrum’ ('waiting room’) for the electronic queue of non-priority-1 healthcare cases awaiting dispatch and/or reassessment. It is not a physical waiting room and was not treated as a separate, universal formal protocol; rather, it denotes the queue-management work described by dispatchers across local settings.

### Participants, sampling and recruitment

Strategic purposive sampling^12 18^ was used to recruit EMDs with experience of prioritisation during periods of constrained ambulance availability. Inclusion criteria were (1) current employment as an ambulance dispatcher in a Swedish EMCC and (2) at least 1 year of dispatch experience. Sampling was operationalised through an evolving recruitment matrix that tracked sex, age, years of dispatch experience, occupational background and broad geographic region. We initially invited dispatchers from several centres and then directed subsequent invitations towards under-represented regions and experience profiles to achieve variation across northern, central and southern Sweden, including metropolitan Stockholm. No patients or members of the public were involved.

With organisational permission, study invitations describing the purpose, procedures and voluntary nature of participation were distributed through internal EMCC communication channels and local managerial contacts. Managers facilitated distribution of the invitation but did not select participants, and interested dispatchers contacted the research team directly. Participation or non-participation had no employment consequences, and managers were not informed which individuals participated.

13 EMDs participated (8 women, 5 men; aged 33–62 years). Because the invitation was distributed internally and non-response was not reported to the research team, the number of employees who received the invitation but chose not to respond and their reasons for non-participation are unknown. No eligible dispatcher who contacted the research team declined after receiving study information, and no participant withdrew or dropped out after consenting. Sample size was guided by information power: the study aim was specific, participants were highly relevant, interviews were in-depth and analysis involved iterative team discussion. Recruitment ceased when no substantively new content emerged that changed the developing category structure.

### Data collection

Data were collected through in-depth semistructured video interviews conducted via Microsoft Teams in May 2025. Interviews were conducted by the first and last author (PH (male) and VV (female)). Both interviewers have professional experience in EMCC/EMS contexts but had no prior relationship with the participants. Only the interviewer and the participant were present. Interviews lasted a mean of 48 min (range 35–62 min). Repeat interviews were not conducted.

The interview guide was developed by the research team based on the study aim, a focused review of literature known to the team from prior work on emergency medical dispatch reporting, dispatch decision-making, EMS response-time modelling and EMS clinical reasoning and operational EMCC/EMS experience.^1 4 5 7 8 11^ The literature was identified through earlier searches conducted for the doctoral project and through citation tracking of key qualitative dispatch and EMS decision-making papers, rather than through a separate systematic review. The guide was discussed within the author team and refined to elicit concrete episodes of scarcity, queue management, geographic trade-offs, information-system use, teamwork and moral strain; it was not pilot tested. It was then used flexibly so participants could lead the narrative. Interviews began with: “Could you tell me about your experiences working with ambulance dispatching during periods of resource shortages?” Probes explored (a) how priorities were determined when multiple patients competed for resources, (b) how queued cases were managed over time, (c) perceived consequences of delay, (d) coordination within the EMCC and with external agencies and (e) workload, stress and coping. The full guide is provided in online supplemental file 1.

All interviews were audio-recorded and transcribed verbatim in Swedish. Transcripts were deidentified and assigned participant numbers. Transcripts were not returned to participants for comment or correction, and no formal participant feedback on preliminary findings was sought. Field notes were written after each interview to capture contextual impressions, emerging topics and reflexive considerations (eg, how interviewer assumptions may have shaped probing). Coding and category development were organised in structured spreadsheets (Microsoft Excel) and text documents.

### Language and translation

Analysis was conducted using Swedish transcripts. Quotations were translated into English by a Swedish English bilingual team member and reviewed by a second bilingual author familiar with EMS terminology. Discrepancies were resolved through discussion with reference to the Swedish original to preserve meaning and nuance.

### Data analysis

An inductive qualitative content analysis was conducted following Elo and Kyngäs.^12^ In line with their description of the preparation phase, PH and VV read each transcript repeatedly to gain a sense of the whole and to understand the context of dispatchers’ experiences. During this familiarisation, we noted preliminary impressions and used field notes to support reflexive awareness of assumptions.

In the organising phase, PH and VV performed open coding line by line. Codes were written close to participants’ wording and without a predefined coding frame. We compared codes for similarities and differences, grouped them into subcategories at a predominantly manifest (text-close) level and then abstracted subcategories into generic categories by capturing shared patterns across participants. Finally, one main category was formulated to express the overarching meaning that integrated the generic categories. Throughout, we moved iteratively between transcripts, codes and evolving categories to refine boundaries and ensure that categories remained grounded in the data.

To distinguish what we did from what the method literature describes, we report our analytic steps (coding, grouping, abstraction, team discussions and audit trail documentation) and cite Elo and Kyngäs^12^ as the methodological source for this analytic approach. Coding sheets and dated versions of category definitions were maintained as part of an audit trail to support dependability and confirmability.

### Trustworthiness and reflexivity

Credibility was supported through purposive sampling of dispatchers with direct experience of scarcity, open-ended interviewing and the use of quotations to demonstrate linkage between data and categories.^12 18^ Analyst triangulation was achieved through iterative analytic meetings, review of coding sheets and category definitions by multiple authors and discussion of alternative interpretations until consensus was reached.^18^

Dependability was strengthened by following a structured content analysis process and documenting analytic decisions over time. Both interviewers’ professional experience in EMCC/EMS contexts supported rapport and context-sensitive probing but also posed a risk of implicit assumptions. To manage this, we used reflexive memoing after interviews and during analysis, explicitly discussed preunderstandings in the author team and invited scrutiny from coauthors with complementary perspectives to challenge interpretations and category boundaries. Confirmability was supported by systematically linking interpretive claims to data extracts and by searching for deviant cases that challenged the emerging structure.^12^ Transferability is supported by a description of the setting and sampling strategy; to protect participant anonymity, detailed site-level characteristics are reported at the level of broad regions rather than specific centres.

### Patient and public involvement

Patients and members of the public were not involved in the design, conduct, reporting or dissemination plans of this research.

## Results

**Figure 1:**
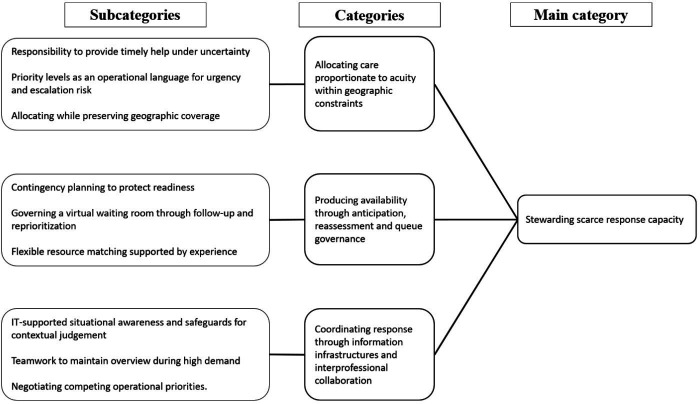
Abstraction pathway from codes → subcategories → generic categories → main category (stewarding scarce response capacity).

The inductive analysis yielded one main category and three generic categories comprising nine subcategories (figure 1). To enhance transparency, we describe how the main category integrates and is supported by the generic categories, and we use quotations selectively to illustrate key patterns.

### Main category: stewarding scarce response capacity

Stewarding scarce response capacity captured dispatchers’ descriptions of sustaining safe service delivery when ambulance availability fluctuated and, at times, was severely constrained. Dispatchers framed their work as stewardship because they were responsible not only for assigning ambulances to individual patients but also for maintaining readiness for unknown future calls. Stewardship involved redistributing risk: delaying some patients in order to preserve capacity for potentially life-threatening events and mobilising or repositioning resources to maintain minimum geographic coverage. The generic categories below describe the key forms of work through which this stewardship was enacted.

### Generic category 1: allocating care proportionate to acuity within geographic constraints

Dispatchers described prioritisation as clinically informed and ethically consequential allocation work carried out in a multipatient environment. Judgements of urgency were made alongside operational constraints, including distance, travel time and the system’s need to preserve coverage for sudden high-acuity incidents. Under scarcity, prioritisation was described as selecting not only who should be dispatched first but also which risks could be accepted at a given moment.

### Responsibility to provide timely help under uncertainty

Dispatchers described a duty to ensure that help was proportionate to need, including anticipating deterioration while patients waited. Ethical reasoning was embedded in routine allocation decisions and intertwined with contextual barriers such as distance, accessibility and uncertainty about the patient’s condition. Participants also described that, during scarcity, striving for an ideal response to every patient could undermine overall safety by exhausting scarce capacity; therefore, they sometimes accepted that lower priorities would exceed nominal time targets in order to protect readiness for time-critical events.

I have the care responsibility. And then I have to make sure the person gets help quickly—how quickly depends on the condition, of course. And where they are… are they out in the forest? Of course that complicates things. (P7)

### Priority levels as an operational language for urgency and escalation risk

Priority levels were described as a shared language that enabled coordination, but dispatchers emphasised that the distribution of priorities across the queue mattered. Cases classified as urgent but not highest priority were described as particularly challenging when multiple such cases accumulated because they required timely response while ambulances were tied up on simultaneous highest-priority assignments. In these situations, dispatchers described weighing nominal targets against contextual indicators of escalation risk (eg, worsening symptoms, safety concerns or prolonged waiting) and the consequences of allocating a unit from far away.

A 2A is, in our world, a priority 1 without blue lights… it’s almost more of a stress factor when several are waiting. (P1)

### Allocating while preserving geographic coverage

Maintaining minimum coverage across a region was described as a recurrent constraint that shaped allocation. Dispatchers weighed whether to dispatch a distant ambulance immediately or to wait for a nearer unit expected to become available soon, recognising that either choice redistributed risk across patients and time. Some described using planned assignments to move ambulances towards areas with poor coverage while keeping them interruptible for escalation. Geographic reasoning was therefore inseparable from clinical prioritisation in situations where the next high-acuity call could arrive at any moment.

You have to think what is actually fastest for the patient: assign an ambulance with a 30-minute drive… or wait for a unit that will soon be available nearby. (P11)

### Generic category 2: producing availability through anticipation, reassessment and queue governance

Dispatchers described actively producing readiness rather than merely responding to incoming calls. This work included contingency planning, continual regulation of a ‘virtual waiting room’ and experience-based strategies to retain control when demand exceeded available ambulances. Readiness was described as a fragile system property that could improve or collapse within minutes, requiring proactive planning and continual reprioritisation.

### Contingency planning to protect readiness

Preparedness was described as continuous replanning and repositioning to protect response capacity as conditions changed. Dispatchers described monitoring where ambulances were likely to become available, anticipating peaks in demand and preparing alternative options (eg, engaging other rescue resources) when ambulances were unavailable. This planning was experienced as essential for meeting time-critical needs despite volatility.

You can’t just have a plan A. You need a plan B, C and so on. (P12)

### Governing a virtual waiting room through follow-up and reprioritisation

Participants described a structured electronic queue for cases that could not be dispatched immediately. Some participants and local routines referred to this queue as a ‘waiting room’ (väntrum); we use ‘virtual waiting room’ to denote this queue-management work rather than a physical location or a uniformly formal national protocol. Governance of this queue involved monitoring, reassessment and reprioritisation to manage clinical risk over time. Dispatchers described relying on reassessment processes (including support from nurses/medical advisors) to detect changes in symptoms and trigger escalation when warranted. They also described how queue length could grow rapidly during simultaneous high-priority events or when ambulances were temporarily unavailable, shifting the work from first-come-first-served dispatch to continuous risk management across waiting patients.

The waiting room is all healthcare calls that are not a priority 1… you check: has anything changed? Do we need to change priority? (P1)

### Flexible resource matching supported by experience

Dispatchers described allocation as matching resource type, competence and response mode to the situation rather than assigning a priority alone. This included deciding when specialised resources were appropriate and when they were too distant to be clinically reasonable. Experience was portrayed as stabilising: it supported pattern recognition, anticipation of bottlenecks and the use of alternative resources when ambulances were unavailable. Experience was also described as increasing tolerance for uncertainty and reducing reactive over-allocation that could destabilise coverage.

It’s experience… you have ambulances, but you also have resources like the fire service… and helicopters. (P7)

### Generic category 3: coordinating response through information infrastructures and interprofessional collaboration

Dispatchers’ stewardship relied on information infrastructures that made resource status visible and on collaboration across roles and agencies to manage concurrency. Participants described coordination as both technical and social: it depended on functional systems that supported situational awareness and on teamwork that distributed tasks and aligned decisions under time pressure.

### Information technology-supported situational awareness and safeguards for contextual judgement

Real-time status monitoring and time tracking supported anticipatory planning by helping dispatchers predict when units would become available and prepare allocations accordingly. Conversely, unexpected time extensions disrupted plans and could cascade across the queue. Dispatchers valued automation and rapid dispatch functions for speed, but emphasised that some call types required contextual listening and should be routed for human judgement to prevent unsafe automatic dispatch.

There are some not included—psychiatry, suicide, assault—where you may need to listen. It’s not good that it goes out automatically directly. (P3)

### Teamwork to maintain overview during high demand

Collaboration within the EMCC helped distribute tasks and maintain shared situational awareness when workload was high. Participants distinguished between formal teamwork built into standard operations, such as involving nurses/medical advisors for reassessment, internal officers for system-level resource coordination or rescue/police roles for joint incidents, and informal peer support in which colleagues monitored queues, answered radio or telephone contacts and helped maintain an overview when the dispatcher responsible for an area was occupied with time-critical tasks.

We collaborate a lot… the ‘loop’ helps… when it’s busy, because you don’t have time to keep up. (P1)

### Negotiating competing operational priorities

Scarcity also created cognitive and moral strain. Participants described stress, frustration and helplessness when patients were waiting and no ambulance was available, particularly when they imagined deterioration, children or a high-acuity event occurring in an uncovered area. The strain was intensified by the fact that dispatchers could redistribute available resources but could not create additional ambulance capacity. Operational negotiations about crew breaks, shift changes and workload distribution were therefore experienced not merely as logistics but as situations in which patient waiting-time risk, staff endurance and system readiness had to be held together.

It stops being enjoyable when you realise that this can affect someone so badly that it becomes a care injury, or in the worst case that someone dies. Then you feel the stress… I have nothing to send. (P1)

Participants described coping by staying task-focused, accepting the limits of their mandate, relying on colleagues and experience and reframing difficult shifts as collective problem-solving. Several contrasted novice anxieties with more experienced pragmatic acceptance: the dispatcher still carried responsibility, but could avoid paralysis by recognising that the safest available action was to do the best possible with the resources present. Peer discussion and informal debriefing after demanding shifts were described as important, although formal support structures were not consistently emphasised.

You cannot tie yourself in knots. I have the resources I have to work with, and I have to do the best I can. (P4)

## Discussion

### Principal findings

This study explored EMDs’ experiences of prioritising patients when ambulance capacity is constrained. Dispatchers described prioritisation under scarcity as stewardship of response capacity: a form of system work that integrates acuity assessment with geographic coverage, queue governance, anticipation, coordination across roles and the management of moral strain. Rather than portraying prioritisation as a linear application of protocol categories, participants described continuously redistributing risk across patients and time while trying to avoid both under-response to urgent need and over-allocation that would compromise readiness for future time-critical events. Interpreted alongside prior work, this supports the view that structured dispatch tools provide a foundation but that experiential and contextual judgement remain central when cases are complex or scarcity intensifies.^11^

### Stewardship as system work and ethical and operational responsibility

The main category, stewardship, captures the dual accountability dispatchers described: responsibility for individual patients in the queue and responsibility for maintaining a functioning emergency response system. This dual accountability rendered prioritisation inherently ethical and operational. Dispatchers described how decisions about who could wait were shaped by judgements about clinical urgency, uncertainty and potential deterioration but also by the recognition that using the ‘last’ available ambulance could increase risk for unknown future calls. In this sense, dispatch prioritisation under scarcity can be understood as system-level clinical reasoning: it integrates structured triage tools with contextual judgement about geography, time and downstream consequences of delay.

Importantly, stewardship was not described as a general attitude but as practical work enacted through specific activities: preserving geographic coverage, monitoring and reprioritising waiting cases, mobilising alternative resources and coordinating with colleagues to maintain overview. Making these activities visible may help move organisational discussions beyond individual ‘good’ or ‘bad’ dispatch decisions to a recognition of the infrastructure and support required to enact safe stewardship.

### Moral strain and coping under structural scarcity

The added analysis of personal strain further specifies stewardship as an ethical and operational responsibility. Dispatchers did not describe strain only as workload or irritation; they described the burden of knowing that delay could harm a patient while also knowing that no additional ambulance could be produced from the dispatch desk. Coping strategies were mainly pragmatic and relational: staying focused on the next safest action, using experience to tolerate uncertainty, relying on colleagues and reframing demanding shifts as successful team performance when harm was avoided. These findings suggest that moral strain is generated by the structural gap between clinical need and available capacity but not simply by individual dispatchers’ decisions. Organisational support should therefore include peer consultation, structured case review and debriefing that recognises scarcity management as safety-critical work rather than treating difficult outcomes solely as individual performance issues.

### Queue governance and reassessment as a safety mechanism

A central contribution of the findings is the depiction of a ‘virtual waiting room’ as an active safety practice rather than a passive backlog. Quantitative and modelling work has examined EMS demand and performance through measures such as response times, workload and system behaviour.^4 5 10^ While such studies improve understanding of time-dependent patterns, they provide limited insight into how scarcity is experienced and managed moment to moment in the dispatch setting. In this study, dispatchers described reassessment and reprioritisation as essential for detecting change over time, particularly when waiting times extend beyond nominal targets. This suggests that patient safety under scarcity depends not only on the initial priority assignment but also on the capacity to sustain follow-up work across waiting patients. Such capacity requires staffing, clear routines and decision-support that makes deteriorating risk visible. Without these, queued patients may become ‘invisible’ and escalation may be detected late. These findings complement existing evidence.^7 8^

### Geographic coverage and equity-oriented trade-offs

Dispatchers’ emphasis on preserving geographic coverage illustrates how equity and readiness constraints enter everyday allocation work. Dispatch and EMS performance literature emphasising timeliness and resource allocation as key system concerns.^1 4 5^ Sending a distant unit may reduce delays for one patient but increase vulnerability for others by leaving areas uncovered. Conversely, waiting for a nearer unit may preserve coverage but prolong the exposure to risk for the patient waiting. These trade-offs were described as unavoidable under scarcity. Organisational guidance that explicitly addresses such trade-offs could support consistency, reduce moral distress and provide a shared language for evaluating decisions when ‘ideal’ responses are not feasible.

### Information infrastructures, automation and collaboration

Dispatchers described information infrastructures as integral to coordination. This is consistent with qualitative dispatch literature emphasising the role of information quality and organisational context in decision-making.^7 8^ Status monitoring and time tracking supported anticipatory planning, while unexpected delays disrupted plans and could cascade across the queue. Participants welcomed automation that increased speed but emphasised that automation must include safeguards for call types that require contextual listening, such as safeguarding concerns or complex psychiatric emergencies. The findings therefore support a principle of ‘selective automation’: rapid functions should be combined with routings that preserve human judgement when nuance is clinically or ethically consequential.

Collaboration within the EMCC and with external agencies functioned as a resilience mechanism. Formal roles supported reassessment, escalation and cross-agency coordination, while informal micro-coordination helped maintain overview when workload was high. However, collaboration also involved negotiating competing operational priorities, and these negotiations were closely linked to dispatcher strain when patients had waited or when crews were fatigued. Interpreted in relation to existing qualitative dispatch evidence, this underscores that coordinated management under scarcity is not only technical but also relational and organisational.^7 8^

### Implications for practice and policy

The implications for practice follow directly from the forms of stewardship described in the results. First, because dispatchers preserved geographic coverage while allocating to individual patients, organisations should make coverage trade-offs visible in routines, supervision and performance review rather than relying solely on aggregate response-time targets. Second, because queued cases require repeated reassessment, training and local routines should explicitly include queue governance, escalation thresholds and handover of waiting patients. Third, because contextual listening was described as essential for complex call types, decision-support and automation should increase speed without bypassing human judgement when nuance is clinically or ethically consequential. Fourth, because participants described moral strain when they could not create capacity, organisations should provide structured peer consultation, debriefing and learning-oriented feedback rather than leaving dispatchers to absorb scarcity decisions individually. Finally, mentorship and structured case review may help newer dispatchers acquire the experience-based judgement described as stabilising under scarcity.

### Strengths and limitations

A strength of this study is the inductive analytic approach, which allowed categories to emerge from dispatchers’ accounts without imposing a predefined framework.^18^ The sample represented multiple regions within the Swedish dispatch system and included variation in age, occupational background and experience. Limitations include voluntary participation, video-based data collection and conducting the study within a single national provider.^9^ Site-level detail has been intentionally limited to protect participant anonymity. Interviewers’ professional EMCC/EMS experience may have influenced data collection and interpretation. Reflexive memoing, documented audit trail work and multiauthor scrutiny were used to mitigate this.^18^

## Conclusion

EMDs described prioritisation under ambulance scarcity as stewardship of response capacity: system work that balances patient acuity, waiting-time risk, geographic readiness, information-system visibility, interprofessional coordination and the moral strain of allocating finite help. The conclusion that dispatchers need organisational structures and decision-support is grounded in four findings: coverage trade-offs were recurrent, the virtual waiting room required sustained reassessment, automation needed safeguards for contextual judgement and personal strain was mitigated mainly through experience and peer support. Strengthening patient safety therefore requires governance that makes queue dynamics and geographic coverage trade-offs visible, protects contextual judgement, sustains follow-up of patients who must wait and supports dispatchers who carry responsibility for scarcity decisions they cannot solve by dispatch action alone.

## Supplementary material

10.1136/bmjopen-2026-118269 online supplemental file 1

## Data Availability

No data are available.
