# Supplementary material for: Stewarding scarce response capacity: an inductive qualitative interview study of emergency medical dispatchers’ prioritising ambulance resources
Source: BMJ Open. 2026 Jul 2;16(7):e118269. doi: 10.1136/bmjopen-2026-118269 (PMC13331129; doi:10.1136/bmjopen-2026-118269)
Supplement: online supplemental file 1 [file bmjopen-16-7-s001.pdf]

Semi-structured interview guide: Emergency medical dispatch under resource scarcity (EMCC)

1) Interview purpose and epistemic stance (for the interviewer)

- **Purpose:** To elicit rich, experience-near accounts of how dispatchers prioritise and coordinate when ambulance resources are scarce, including how they manage queues, trade-offs, collaboration, and information infrastructures.
- **Approach:** Inductive, open-ended, non-evaluative. Encourage *narratives* and *concrete episodes*; use probes to deepen detail rather than to confirm categories.
- **Key interviewing behaviours:** neutrality, curiosity, “tell me more,” ask for examples, clarify meanings of local terms.

2) Pre-interview procedures (10–15 min, before recording)

Eligibility check

- Current role in ambulance dispatch / EMCC function.
- Minimum experience threshold (adapt to protocol).

Logistics

- Confirm quiet setting, stable connection, estimated duration.
- Explain recording, transcription, de-identification, and right to pause/stop.

Consent confirmation (verbal)

- Participation voluntary; can decline any question; can withdraw during interview.
- How data will be stored/used; confidentiality limits (if any).

3) Opening script (read verbatim; 2–3 min)

"Thank you for participating. I am interested in your experiences of dispatch work during periods when ambulance resources are limited. There are no right or wrong answers; I want to understand how you experience and handle these situations in practice. With your permission, I will audio-record the interview to ensure accuracy. You can pause or stop at any time and you may choose not to answer any question. Do I have your permission to record?"

4) Warm-up and contextual background (5–10 min)

1. **Role and experience**
    - "Could you briefly describe your current role and typical tasks during a shift?"
    - Probes: years in dispatch; prior clinical background; typical shift pattern; main responsibilities (call-taking vs dispatch vs coordination).
2. **Work context**
    - "How would you describe the context you work in (e.g., volume, geography, typical staffing, resources available)?"
    - Probes: urban/rural mix; collaboration roles (nurse/medical advisor, internal officer, rescue desk).

5) Core narrative: experience of scarcity (10–15 min)

Anchor question (start broad, then narrow):

1. "Could you tell me about your experiences working with ambulance dispatching during periods of resource shortages?"
    - Prompts:
        - "What does 'resource shortage' look like in your setting?"
        - "How do you notice that the system is becoming strained?"
        - "What tends to happen first when capacity becomes insufficient?"

Episode elicitation (increase specificity):
2. "Please think of a recent shift or situation when resources were scarce. Can you walk me through what happened from start to finish?"

- Probes:
    - triggers (queue build-up, lack of available units, geography gaps)
    - what information you had at the time
    - key decision points and changes over time

6) Prioritisation and allocation under constraints (15–20 min)

3. **Clinical–operational prioritisation**
   - "How do you determine who needs help first when multiple calls compete for limited resources?"
   - Probes:
     - use of triage/protocols vs contextual judgement
     - how you interpret "acuity" in practice
     - situations where the 'priority level' feels insufficient to describe urgency
4. **Geography, coverage, and readiness**
   - "How do geographic constraints and the need to maintain coverage influence your dispatch decisions?"
   - Probes:
     - keeping preparedness for time-critical events
     - sending resources from further away vs holding them for coverage
     - cross-border/cross-area collaboration; thresholds for borrowing resources
5. **Ethical dimensions and responsibility**
   - "When you decide that someone must wait, how do you think about responsibility and fairness?"
   - Probes:
     - what feels ethically difficult
     - how you judge "safe to wait"
     - how you manage uncertainty about deterioration

7) Managing the queue and reassessment ("virtual waiting room") (15–20 min)

6. **Queue governance**
   - "Can you describe how waiting cases are handled when you cannot dispatch immediately?"
   - Probes:
     - how cases enter the queue; visibility/overview
     - how often and how reassessment occurs
     - reprioritisation: triggers, thresholds, and practical constraints
7. **Safety practices during waiting**
   - "What safeguards exist for patients who are waiting, and how do you decide when to escalate?"
   - Probes:
     - call-backs, monitoring routines, decision to re-contact
     - warning signs you listen for (clinical and contextual)
     - documenting changes; communicating risk within the team

8) Preparedness, adaptability, and experience (10–15 min)

8. **Preparedness and anticipatory planning**
    - "How do you plan ahead during a shift to maintain readiness when you expect scarcity?"
    - Probes:
        - contingency plans (plan B/C)
        - redistribution or staging logic
        - how you balance immediate demand against future unknown calls
9. **Experience as a resource**
    - "In what ways does experience influence how you manage scarce resources?"
    - Probes:
        - recognition patterns; 'sense of control'
        - knowledge of alternative pathways/resources
        - learning moments: what you wish you had known earlier

9) Information infrastructures and decision support (10–15 min)

10. **IT systems and situational awareness**

- "How do information systems support (or hinder) your ability to coordinate during scarcity?"
- Probes:
    - status monitoring; time tracking; visibility of unit availability
    - points where system delays/disruptions affect plans
    - workarounds when systems are degraded

11. **Automation and decision support**

- "Are there functions that automate or accelerate dispatching? How do you experience these during busy periods?"
- Probes:
    - when automation helps
    - when human listening/judgement is essential
    - perceived safety risks; safeguards you would want

10) Collaboration and coordination (10–15 min)

12. **Intra- and interprofessional collaboration**

- "Who do you collaborate with most during resource shortages, and how does collaboration influence decisions?"
- Probes:
  - coordination with internal officers, nurses/medical advisors, rescue services, ambulance crews
  - teamwork practices that improve overview during peaks
  - misalignment of priorities; how conflicts are resolved; consequences for patients

11) Impact on the dispatcher (optional but recommended; 8–12 min)

13. **Workload, stress, and moral strain**

- "How does prolonged scarcity affect you during and after shifts?"
- Probes:
  - stressors (cognitive load, moral pressure, uncertainty)
  - coping strategies; peer support; supervision
  - debriefing routines; what support is missing

12) Improvement-oriented questions (8–12 min)

14. **Training and organisational support**

- "What would most improve your ability to prioritise safely during scarcity?"
- Prompts:
  - training (queue management, reassessment, escalation)
  - staffing/roles; supervision
  - information tools; feedback loops; learning from outcomes

15. **Policy and system design**

- "If you could change one thing in the system to reduce risk when resources are scarce, what would it be—and why?"
- Probes:
  - response capacity; coverage logic
  - collaboration structures; escalation pathways
  - metrics that matter (beyond response times)

13) Optional vignettes (use only if time allows; 10–15 min)

Vignette A: concurrent competing demands

- “Imagine two urgent calls arrive within minutes: one suspected time-critical medical emergency; another potentially unstable psychiatric/suicidality case. Only one ambulance is immediately available nearby. How would you reason through this situation?”
    - Probes: information you seek first; trade-offs; alternative resources; collaboration; what would change your decision.

Vignette B: prolonged waiting and uncertainty

- “A low-priority case has waited a long time. The initial information suggested low risk, but time is passing and the queue is growing. How do you decide whether to reassess or escalate?”
    - Probes: triggers; time thresholds (if any); communication routines; documentation.

(Emphasise: “This is not a test; I am interested in your reasoning and the constraints you work within.”)

14) Closing (3–5 min)

- “Is there anything important about prioritisation during scarcity that we have not discussed?”
- “If you were advising a new dispatcher about handling shortages, what would you tell them?”
- “Do you have any questions for me?”
- Thank participant; reiterate confidentiality; explain next steps (transcription, de-identification).

Semi-strukturerad intervjuguide guide: Emergency medical dispatch under resource scarcity (EMCC)

1) Syfte och intervjuarhållning (för intervjuaren)

- **Syfte:** Att generera rika, erfarenhetsnära berättelser om hur larmoperatörer/dirigenter prioriterar och koordinerar när ambulansresurser är otillräckliga, inklusive köhantering, avvägningar, samverkan och informationsstöd.
- **Intervjuansats:** Induktiv och icke-värderande. Eftersträva konkreta händelseförlopp, beslutspunkter och resonemang.
- **Teknik:** Be om exempel, följ tidslinjen (“vad hände sedan?”), klargör begrepp (“vad betyder det för dig?”).

2) Förberedelser före inspelning (10–15 min)

- Kontrollera att deltagaren kan tala ostört, och att tekniken fungerar.
- Informera om:
  - inspelning och transkribering,
  - avidentifiering,
  - frivillighet och rätt att avstå frågor/avbryta.
- Bekräfta samtycke (muntligt) och starta inspelning.

3) Inledande manus (läs gärna ordagrant; 2–3 min)

“Tack för att du deltar. Jag är intresserad av dina erfarenheter av ambulansdirigering under perioder när resurserna inte räcker till. Det finns inga rätt eller fel svar; jag vill förstå hur du upplever och hanterar sådana situationer i praktiken. Med ditt tillstånd kommer jag att spela in intervjun för att säkerställa korrekthet. Du kan när som helst pausa eller avbryta och du kan avstå från att besvara enskilda frågor. Är det okej att jag spelar in?”

4) Uppvärmning och bakgrund (5–10 min)

1. **Roll och erfarenhet**
   - "Kan du beskriva din nuvarande roll och dina huvudsakliga arbetsuppgifter under ett pass?"
   - Prober: antal år i funktionen; tidigare klinisk bakgrund; arbetssätt (samtal/dirigering/koordinering).
2. **Arbetskontext**
   - "Hur skulle du beskriva kontexten där du arbetar (t.ex. belastning, geografi, bemanning, resurser)?"
   - Prober: variation över dygnet/veckan; toppar; vanliga flaskhalsar.

5) Kärnberättelse: att arbeta vid resursbrist (10–15 min)

3. **Öppen ingång**
   - "Berätta om dina erfarenheter av att arbeta när ambulansresurserna är otillräckliga."
   - Prober:
     - "Hur märks det att systemet blir ansträngt?"
     - "Vad tenderar att hända först när kapaciteten inte räcker?"
4. **Konkreta exempel (händelseförlopp)**
   - "Tänk på ett nyligt pass eller en situation med resursbrist. Kan du gå igenom vad som hände, steg för steg?"
   - Prober:
     - utlösande faktorer,
     - vilka informationskällor som fanns,
     - centrala beslutspunkter och förändringar över tid.

6) Prioritering och resursallokering under begränsningar (15–20 min)

5. **Prioriteringslogik**
   - "Hur avgör du vem som behöver hjälp först när flera ärenden konkurrerar om få resurser?"
   - Prober:
     - protokoll/beslutsstöd vs professionell bedömning,
     - osäkerhet och risk,
     - när prioriteringsnivåer känns otillräckliga för att beskriva 'brådska'.
6. **Geografi, täckning och beredskap**
   - "Hur påverkar geografi och behovet av att upprätthålla täckning dina beslut?"
   - Prober:
     - skicka en enhet långt bort vs behålla beredskap,
     - prioritering av framtida okända larm,
     - samverkan över områdesgränser.

7. **Etiska avvägningar och ansvar**
    - “När du behöver fatta beslut som innebär att någon får vänta—hur resonerar du kring ansvar, rimlighet och rättvisa?”
    - Prober:
        - situationer som upplevs etiskt svåra,
        - ‘säker väntan’ och tecken på försämring,
        - hur du hanterar att du inte kan veta utfallet.

7) Köhantering och omprövning (”virtuell väntrumshantering”) (15–20 min)

8. **Hur kön fungerar i praktiken**
    - “Hur hanteras ärenden som inte kan få ambulans direkt?”
    - Prober:
        - hur ärenden hamnar i kö,
        - överblick och synlighet,
        - dokumentation och spårbarhet.
9. **Omprövning och omprioritering**
    - “Hur går omprövning till när väntetiden blir lång eller ny information tillkommer?”
    - Prober:
        - återuppringning/rutiner,
        - trösklar för eskalering,
        - vad som praktiskt hindrar omprövning.
10. **Patientsäkerhetsstrategier under väntan**

- “Vilka säkerhetsmekanismer använder ni för patienter som väntar, och när väljer du att eskalera?”
- Prober:
    - varningssignaler (kliniska och kontextuella),
    - samråd med kollegor/medicinsk rådgivning,
    - hur risk kommuniceras internt.

8) Beredskap, anpassning och erfarenhet (10–15 min)

11. **Anticipatorisk planering**

- “Hur planerar du under ett pass när du förväntar dig resursbrist?”
- Prober:
    - plan B/C,
    - strategier för omfördelning och positionering,
    - balansering mellan akut behov och framtida beredskap.

12. **Erfarenhet som resurs**

- "På vilket sätt påverkar erfarenhet hur du hanterar resursbrist?"
- Prober:
    - mönsterigenkänning,
    - hantering av osäkerhet,
    - viktiga lärdomar.

9) Informationsstöd och teknik (10–15 min)

13. **Systemstöd och situationsmedvetenhet**

- "Hur stödjer (eller hindrar) informationssystem och verktyg ditt arbete vid hög belastning?"
- Prober:
    - överblick över resurser och status,
    - tidsspårning och prioriteringssignalering,
    - workaround-lösningar vid systembegränsningar.

14. **Automatisering och beslutsstöd**

- "Finns det automatiserade funktioner eller beslutsstöd som påverkar dirigeringen? Hur fungerar det vid resursbrist?"
- Prober:
    - när det hjälper,
    - när mänsklig bedömning är avgörande,
    - upplevda risker och skyddsmekanismer.

10) Samverkan och koordinering (10–15 min)

15. **Samverkan i och utanför organisationen**

- "Vilka samarbetar du mest med under resursbrist, och hur påverkar samverkan besluten?"
- Prober:
    - koordinerande roller,
    - kommunikation med ambulansbesättningar,
    - oenighet om prioriteringar och hur det hanteras.

11) Påverkan på individen (valfri men rekommenderad; 8–12 min)

16. **Belastning, stress och moralisk press**

- "Hur påverkar långvarig resursbrist dig under och efter passen?"
- Prober:
  - kognitiv belastning,
  - moralisk stress,
  - stöd, återhämtning, debriefing.

12) Förbättringar och lärande (8–12 min)

17. **Förbättringsförslag**

- "Vad skulle mest förbättra möjligheten att prioritera säkert under resursbrist?"
- Prober: utbildning, bemanning/roller, återkoppling, systemstöd.

18. **Systemdesign**

- "Om du fick förändra en sak i systemet för att minska risk vid resursbrist—vad skulle du ändra och varför?"
