# Supplementary material for: Stewarding scarce response capacity: an inductive qualitative interview study of emergency medical dispatchers’ prioritising ambulance resources
Source: BMJ Open. 2026 Jul 2;16(7):e118269. doi: 10.1136/bmjopen-2026-118269 (PMC13331129; doi:10.1136/bmjopen-2026-118269)
Supplement: Supplementary file 2 [file bmjopen-2026-118269.reviewer_comments.pdf]

PEER REVIEW HISTORY

BMJ Open publishes all reviews undertaken for accepted manuscripts. Reviewers are asked to complete a checklist review form and are provided with free text boxes to elaborate on their assessment. These free text comments are reproduced below.

ARTICLE DETAILS

Title (Provisional)

Stewarding scarce response capacity: an inductive qualitative interview study of emergency medical dispatchers prioritising ambulance resources

Authors

Hill, Peter; Lederman, Jakob; Jonsson, Daniel; Bolin, Peter; Vicente, Veronica

---

VERSION 1 - REVIEW

**Reviewer**	**1**
**Name**	**Schmiedhofer, Martina**
**Affiliation**	**Charité Universitätsmedizin Berlin, Emergency and Acute Medicine**
**Date**	**17-Apr-2026**
**COI**	**None**

Thank you very much for inviting me to review this manuscript, which addresses an important topic in emergency care: the deployment of ambulances, taking into account availability, distances and the presumed urgency of care. The manuscript provides a well-written and clear introduction to the research topic, starting with a detailed presentation of the national emergency system in which the research is being conducted. This allows readers of an international journal to interpret the results within the relevant national organisational context. Though, to further improve the quality of the manuscript, there are some comments.

The composition of the sampling is described, but lacks the recruitment strategy: how was the target group adressed and were there any rejections to participate and if so, for what reason?

The results are well structured and provide a very good insight into the day-to-day demands and the flexibility required to meet them as effectively and safely as possible. The unpredictability of the number of help seeking patients, their regional distribution and level of acuity may raise concerns about patient safety and the prevention of unnecessary harm caused due to the burden of of longer waiting times amd/or the deterioration of the current condition. This may lead to moral stress, as the dispatchers have no impact on the provision

of additional resources. Against this backdrop, some more attention should be cgiven to the personal strains and how the participants are coping with. These aspects are included in the semi-structured questionnaire. However, in the results they only appear in the final section with mild annoyance regarding crew members’ break times as ‚negotiating competing operational priorities‘, against delayed operations. If the interview material contains more statements or reflections on the psychological burden caused by a lack of amublances and/or staff, this, it would be a valuable addition to the presented results.

Reviewer **2**

Name **Lindner, Thomas**

Affiliation **Stavanger University Hospital, Regional competency center for emergency medicine (RAKOS)**

Date **11-May-2026**

COI **None**

Thank you for your work entitled: “Stewarding scarce response capacity: an inductive qualitative interview study of emergency medical dispatchers prioritising ambulance resources.” I have several remarks that I would like to bring to your attention.

Setting

The description of the setting should be expanded. How many EMCCs are there in Sweden, and what are their catchment areas? Are there alternative emergency care numbers or pathways that patients may use? Please describe the Swedish EMCC landscape in greater detail, including dispatcher educational level/background, dispatch protocols used, and the definition of the different priority levels.

Sampling and variation

You state: “We sought variation in sex, age, experience and broad geographic region (northern, central and southern Sweden, including metropolitan Stockholm) to support transferability.” Please explain more clearly how this variation was achieved and operationalised during recruitment.

Data collection

In the Methods section, you write that the interview guide was informed by “relevant literature on emergency medical dispatch and operational experience.” Please specify which literature was used and how it was identified.

Teamwork

In the Results section, you describe teamwork between dispatchers. However, it is unclear how dispatch teams are usually organised. Is teamwork part of standard operational

procedure, supported by protocols, or does it occur more exceptionally or informally? This should be clarified in the Setting section.

"Virtual waiting room"

The term "virtual waiting room" requires clarification. Is this an established concept, part of a formal protocol, or an informal expression used by some dispatchers? Please explain the origin and meaning of this term within the paper.

Conclusion

In the conclusion, you state: "Supporting dispatchers' stewardship requires organisational structures and decision-support that make trade-offs visible, protect contextual judgement and sustain follow-up of patients who must wait." For me, it is not entirely clear how this conclusion is directly supported by the presented results. Please strengthen the link between the findings and this concluding statement.

VERSION 1 - AUTHOR RESPONSE

Reviewer 1

Comment 1: Recruitment strategy and non-participation

Response: We agree that the recruitment process needed greater transparency. The Methods now specify how strategic purposive sampling was operationalised through an evolving recruitment matrix tracking sex, age, dispatch experience, occupational background and broad geographic region. We also clarify how invitations were distributed, that managers facilitated distribution but did not select participants, that interested dispatchers contacted the research team directly, and that participation or non-participation had no employment consequences. We now report that the number of non-responders and their reasons are unknown because the invitation was distributed internally, and that no eligible dispatcher who contacted the research team declined after receiving study information or withdrew after consent.

Manuscript change/location: Participants, sampling and recruitment, lines 248-286.

Comment 2: Personal strain and coping under ambulance/resource shortage

Response: We re-examined the material for accounts of psychological burden, moral strain and coping. The Results section has been expanded under the subcategory "Negotiating competing operational priorities." The revised text now reports stress, frustration and helplessness when patients were waiting and no ambulance was available, including dispatchers' concerns about deterioration, children or high-acuity events occurring in uncovered areas. We added participant quotations and a new paragraph on coping: staying task-focused, accepting the limits of the dispatch mandate, relying on colleagues and experience, and using informal peer discussion or debriefing. The added analysis has also been integrated into the abstract, Discussion, implications and Conclusion so that strain is treated as part of stewardship rather than as an isolated issue.

Manuscript change/location: Abstract, lines 47-68; Results, lines 534-568; Discussion, lines 612-624; Implications, lines 675-700; Conclusion, lines 713-724.

Reviewer 2

Comment 1: Expanded Setting

Response: We have substantially expanded the Setting section. It now describes the Swedish emergency medical dispatch system, SOS Alarm's role, the number and geographic spread of SOS centres, the national handling of 112 call-taking and local handling of ambulance dispatch/rescue operations, catchment areas, cross-border coordination, and alternative urgent care pathways. We also added a functional description of priority levels 1A, 1B, 2A, 2B, 3 and 4/lying medical transport, clarified that exact response-time targets and resource rules vary by region, and described decision support, dispatcher backgrounds, internal training and EMCC team organisation. The reference list has been corrected and updated to support these setting claims.

Manuscript change/location: Setting, lines 182-231; references 14-17, lines 779-790.

Comment 2: Sampling and variation

Response: We clarified how variation was achieved. The revised Methods now state that variation was operationalised through an evolving recruitment matrix and that subsequent invitations were directed towards under-represented regions and experience profiles as recruitment progressed. We also added a more explicit description of the sample and non-participation, including sex, age range, non-response handling, withdrawals/drop-outs and information power.

Manuscript change/location: Sampling strategy, lines 248-260; recruitment process, lines 262-271; sample description, non-participation and information power, lines 274-286.

Comment 3: Literature informing the interview guide

Response: We agree that the basis for the interview guide should be clearer. The revised Data collection section now specifies that the guide was based on the study aim, a focused review of literature known to the team from prior work on emergency medical dispatch reporting, dispatch decision-making, EMS response-time modelling and EMS clinical reasoning, and operational EMCC/EMS experience. We clarify that the literature was identified through earlier doctoral-project searches and citation tracking of key qualitative dispatch and EMS decision-making papers, rather than through a separate systematic review. We also state how the guide was refined and that it was not pilot tested.

Manuscript change/location: Data collection, lines 289-327, particularly the interview-guide development and literature-identification description at lines 297-311; relevant literature references 1, 4, 5, 7, 8 and 11, lines 734-771.

Comment 4: Teamwork and team organisation

Response: We clarified both the general team organisation and how teamwork operated in the data. In the Setting section, teamwork is now described as partly formalised through roles, shared information systems, call-monitoring functions and regional routines, and partly informal through peer-to-peer micro-coordination during high workload. In the Results, participants' descriptions now explicitly distinguish formal teamwork built into standard operations from informal peer support that helped maintain situational awareness when workload was high. The

Discussion links collaboration to resilience while noting that it also involved difficult operational negotiations.

Manuscript change/location: Setting, lines 215-224; Results, lines 517-528; Discussion, lines 651-674.

Comment 5: Clarification of "virtual waiting room"

Response: We clarified the origin and meaning of the term. The Setting now explains that "virtual waiting room" is our analytic shorthand grounded in participants' use of the Swedish term "väntrum" for the electronic queue of non-priority-1 healthcare cases awaiting dispatch and/or reassessment. We explicitly state that it is not a physical waiting room and was not treated as a separate, universal formal protocol. The Results section further clarifies that the concept denotes queue-management work involving monitoring, reassessment and reprioritisation across waiting patients.

Manuscript change/location: Definition in Setting, lines 225-231; Results, lines 462-480; Discussion, lines 626-639.

Comment 6: Link between findings and conclusion

Response: We strengthened the link between the results and the conclusion. The Discussion now presents stewardship as practical work enacted through preserving geographic coverage, monitoring and reprioritising waiting cases, mobilising alternative resources and coordinating with colleagues. The Implications section now explicitly follows from four forms of stewardship in the Results: coverage trade-offs, queue governance, contextual judgement in automation, and moral strain. The Conclusion now states that the need for organisational structures and decision support is grounded in these four findings rather than presenting this as an unsupported general recommendation.

Manuscript change/location: Principal findings and stewardship discussion, lines 570-610; moral strain discussion, lines 612-624; Implications, lines 675-700; Conclusion, lines 713-724.

Additional checks completed

COREQ alignment was rechecked across all 32 items. The revised manuscript now reports interviewer identity, credentials, gender, experience, prior relationship, recruitment method, non-participation, setting, sample description, interview guide, repeat interviews, recording, field notes, duration, information power, transcript return, number of coders, inductive coding, analysis process, software, participant checking, quotations and the relationship between data and findings.

Reference checking was also performed for the new or revised setting and methods claims. The revised reference list now includes official sources for SOS Alarm's 112 role and the Swedish EMCC landscape, 1177/112 advice, and ambulance prioritisation and dispatch (references 14-17, lines 779-790), plus Elo and Kyngäs for qualitative content analysis and Thomas for the general inductive approach (references 12 and 18, lines 772-778 and 791-792).

VERSION 2 - REVIEW

Reviewer 2

Name **Lindner, Thomas**

Affiliation **Stavanger University Hospital, Regional competency center for emergency medicine (RAKOS)**

Date **15-Jun-2026**

COI

Thank you for the revised manuscript. You have addressed my suggestions sufficiently.
